# Treatment of Autoimmune Pancreatitis with the Anecdotes of the First Report

**DOI:** 10.1155/2012/597643

**Published:** 2012-04-03

**Authors:** Terumi Kamisawa, Tadashi Takeuchi

**Affiliations:** ^1^Department of Internal Medicine, Tokyo Metropolitan Komagome Hospital, Tokyo 113-8677, Japan; ^2^Pancreas Research Foundation of Japan, Tokyo 164-0011, Japan; ^3^Department of Gastroenterology, Tokyo Women's Medical University, Tokyo 162-8666, Japan

## Abstract

The first case that led researchers to put forward a new concept of autoimmune pancreatitis (AIP) was treated with steroids by gastroenterologists in Tokyo Women's Medical University. It is important to differentiate AIP from pancreatic cancer before treatment with steroids is started. Today, steroids are standard therapy for AIP worldwide. In the Japanese consensus guidelines, steroid therapy is indicated for symptomatic AIP. After management of glucose levels and obstructive jaundice, oral prednisolone is initiated at 0.6 mg/kg/day for 2–4 weeks and is gradually tapered to a maintenance dose of 2.5–5 mg/day over 2-3 months. To prevent relapse, maintenance therapy with low-dose prednisolone is used. For relapsed AIP, readministration or increased doses of steroids are effective. The presence of proximal bile duct stenosis and elevated serum IgG4 levels may be predictive of relapse of AIP. It is necessary to verify the validity of the Japanese regimen of steroid therapy for AIP. The necessity, drugs, and duration of maintenance therapy for AIP need to be clarified by prospective studies.

## 1. Introduction

The first case that led researchers to put forward a new concept of autoimmune pancreatitis (AIP) was treated with steroids by gastroenterologists (Professor Tadashi Takeuchi) in Tokyo Women's Medical University, and the concept was proposed by Yoshida, a member of the group, in 1995 [1]. This paper describes the anecdotes of treatment of the first case that led researchers to put forward a new concept of AIP and reviews current strategies for treatment of this disorder.

## 2. Anecdotes of Treatment of the First AIP Case

In 1993, a 68-year-old woman who had undergone exploratory laparotomy for jaundice and an abdominal tumor at another hospital, but she was found to have advanced pancreatic cancer that was inoperable, and one month after being discharged she came to Tokyo Women's Medical University Hospital to be treated for pancreatic cancer.

Her general condition was good, and during that 1-month period her jaundice had spontaneously improved without any treatment. Based on the physical examination, the laboratory results, and the findings obtained by diagnostic imaging, AIP would come to mind today, but there was no concept of AIP in those days.

As far as steroid therapy is concerned, we have to pay attention to side effects or complications such as steroid-induced pancreatitis. However, it was fortunate that steroid therapy was dramatically effective without any side effects in this patient; her physical findings, laboratory data, and diagnostic imaging findings became normal, and she was discharged uneventfully.

The 8-week steroid therapy was effective, with the results that hyperglobulinemia and positive autoantibody were normalized, and swelling of the pancreas and irregular narrowing of the main pancreatic duct were also normalized. Those were clearly attributable to an autoimmune mechanism, and we proposed autoimmune pancreatitis.

By the way, the case report was submitted to* Digestive Diseases and Sciences *in the summer of 1993 [1], and it had taken about two years since it had originally been submitted. There was a comment by a reviewer that since it was just a report of a single case, adding the subtitle “Proposal of the Concept of Autoimmune Pancreatitis” may have been an overstatement or an exaggeration. However, retaining the subtitle made a considerable impact, and it might have attracted a great deal attention in the age of the Internet. Obviously, it is difficult to propose anything new.

## 3. Treatment of AIP

AIP has recently been subclassified into type 1 and type 2 AIP [2, 3]. Type 1 AIP is a classical AIP that shows a histology of lymphoplasmacytic sclerosing pancreatitis and is considered the pancreatic manifestation of IgG4-related systemic disease [4, 5]. Type 2 AIP shows a histology of idiopathic duct-centric chronic pancreatitis and is not related to IgG4 [2, 3]. Although it is reported that type 2 AIP responds well to steroid therapy, similar to type 1 AIP [2], the precise clinical features of type 2 AIP have not been clarified. Only treatment of type 1 AIP is described in this paper.

### 3.1. Spontaneous Improvement

In some AIP patients, improvement of swelling of the pancreas or irregular narrowing of the main pancreatic duct improves without steroid therapy [6–8]. In our series [6], 3 of 12 AIP patients who were followed for more than 6 months without steroid therapy improved spontaneously, but 2 of them received steroid therapy later. Wakabayashi et al. [7] reported on 4 AIP patients who showed spontaneous regression; they had negative immunoserological tests and no biliary lesions. Kubota et al. [8] reported that seronegative findings for IgG4, no obstructive jaundice, and focal swelling of the pancreas were related to spontaneous remission. Considering that patients who are later treated with steroids due to AIP exacerbation also show steroid responsiveness, asymptomatic segmental AIP cases without biliary lesions may be followed without steroid therapy with periodic laboratory and imaging tests.

### 3.2. Indication

Because the fibroinflammatory process in AIP responds well to steroid therapy, administration of oral steroids has become standard therapy for AIP. However, it is important to differentiate pancreatic cancer from AIP before starting treatment with steroids in AIP patients. According to a recent international study of AIP [9], steroids are used for AIP patients in all countries. In the Japanese consensus guidelines for the management of AIP [10], indications for steroid therapy in AIP include symptoms such as obstructive jaundice due to associated sclerosing cholangitis and the presence of symptomatic extrapancreatic lesions such as hydronephrosis due to retroperitoneal fibrosis. Because diabetes mellitus (DM) seen in the acute presentation of AIP sometimes improves with steroid therapy, DM coincidental with AIP might be an indication for steroid therapy [10]. Although improvement in clinical findings with steroid therapy may be useful in the differential diagnosis of AIP from pancreatic cancer, facile diagnostic steroid trial should be avoided not to misdiagnose pancreatic cancer as AIP. Diagnostic steroid trials should be conducted carefully by pancreatologists only after a negative workup for cancer including endoscopic ultrasound-guided fine needle aspiration [11, 12]. Serological and imaging tests should be done 2 weeks after commencement of steroid therapy. Rapid response to steroids is reassuring and confirms the diagnosis of AIP. If steroid effectiveness is reduced, the patient should be reevaluated on suspicion of pancreatic cancer.

### 3.3. Steroid Regimen

**Figure 1:**
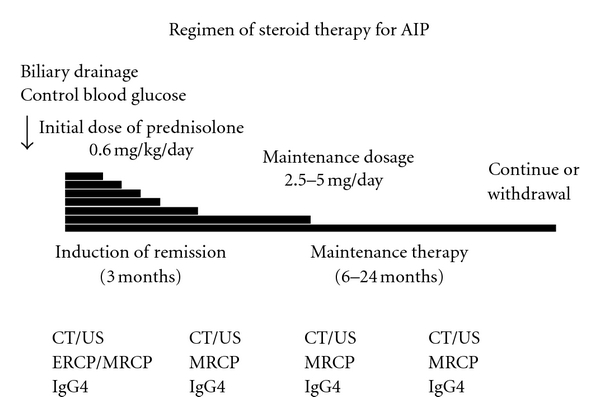
Regimen of oral steroid therapy for AIP.

In the Japanese guidelines [10], before starting steroid therapy, biliary drainage is usually done in cases with obstructive jaundice. However, as there are some patients whose jaundice is relieved by steroid therapy alone, it is unclear if biliary obstruction can be treated with steroid therapy alone without biliary drainage [13]. In cases with DM, glucose levels must be controlled. The recommended initial oral prednisolone dose is 0.6 mg/kg/day. Serological and imaging tests should be done periodically after commencement of steroid therapy [10]. Magnetic resonance cholangiopancreatography is useful to observe the response to steroids in the pancreaticobiliary ducts noninvasively [14]. Pancreatic size usually normalizes within a few weeks, and biliary drainage becomes unnecessary within about 1 month. Rapid response to steroids is reassuring and confirms the diagnosis of AIP. If steroid effectiveness is reduced, the patient should be reevaluated with a suspicion of pancreatic cancer. The initial dose of steroids should be administered for 2–4 weeks, and the dose should be gradually tapered to a maintenance dose of 2.5–5 mg/day over 2-3 months [10] (Figure 1).

In the Mayo Clinic [15], prednisolone is used at 40 mg/day for 4 weeks and is tapered by 5 mg/week for a total of 11 weeks of therapy. In Korea [16], remission is achieved on a regimen of prednisolone 0.5 mg/kg per day for 1-2 months followed by a gradual tapering of 5–10 mg per month to a maintenance dose of 2.5–7.5 mg/day.

### 3.4. Remission

Remission is defined as the disappearance of clinical symptoms and resolution of the pancreatic and/or extrapancreatic manifestations on imaging studies [17].

In a multicenter survey of steroid therapy for AIP [17], at remission, the enlarged pancreas returned to near-normal size in 239 of 300 patients (80%) and became atrophic in 58 patients (20%). Elevated serum IgG4 levels decreased in all patients after the start of steroid therapy but failed to normalize (<135 mg/dL) in 115 of 182 patients (63%). At remission, irregularity of the pancreatic ducts and/or some degree of bile duct stenosis remained in 67 of 115 patients (58%) with persistent elevation of serum IgG4 levels, but only 18 of 67 patients (27%) with normalized serum IgG4 levels.

In our other study [18], HbA1c decreased by more than 0.5% in 8 of 21 AIP patients (38%) with DM after 3 months of steroid therapy. One year after the start of therapy, HbA1c decreased compared with levels before steroid therapy in 13 of 15 DM patients (87%). Impaired pancreatic exocrine function improved in all AIP patients and normalized in half of them [19].

### 3.5. Relapse and Maintenance Therapy

Relapse of AIP is defined as reappearance of symptoms with the reappearance of pancreatic and/or extrapancreatic (including bile duct, salivary gland, and retroperitoneum) abnormalities on imaging and/or elevation of serum IgG4 levels [17].

In a multicenter survey [17], the relapse rate of AIP patients was significantly lower in those who received steroid therapy (24%, 110/451) than in those not given steroid therapy (42%, 32/77). There was no correlation between the relapse rate and the initial prednisolone dose (40 mg/day, 19% (31/160) versus 30 mg/day, 23% (65/283)). In patients who received steroid therapy, relapse occurred in the pancreas (*n* = 57, 52%), bile duct (*n* = 37, 34%), and extrapancreatic lesions (*n* = 19; salivary gland swelling (*n* = 10), interstitial pneumonia (*n* = 4), and others (*n* = 5)).

Maintenance steroid therapy (oral prednisolone dose: 2.5 mg–5 mg/day) was given after remission in 377 of 459 patients (82%) treated with steroids. The relapse rate with maintenance therapy was 23% (63/273), which was significantly lower than that of patients who stopped maintenance therapy (34%, 35/104) [17]. In the United States and United Kingdom, where no maintenance therapy was given, relapse rates of patients treated with steroids were reportedly 38–60% [20–22]. In Korea, where maintenance therapy was stopped completely after about 6 months, the relapse rate of AIP patients treated with steroids was 33% (13/40) [16]. In consideration of these findings, maintenance therapy with low-dose prednisolone may prevent relapse. In Japan, maintenance therapy is used for about 1–3 years. However, the optimal duration of maintenance therapy is an issue requiring further investigation, as continued steroid therapy may increase the risk of steroid-induced adverse events. AIP often occurs in the elderly, who are already at heightened risk for osteoporosis and complications of glucose intolerance.

For relapsed AIP, readministration or increasing the dose of steroids is effective. In the United States and United Kingdom, immunomodulatory drugs such as azathioprine were used for maintenance of remission in patients with relapse after steroid withdrawal although azathioprine also has adverse effects such as allergic reactions, bone marrow suppression, and increased risk of infection [20–22]. Recently, it was reported that an AIP patient refractory to steroids and 6 mercaptopurine was successfully treated with rituximab, a monoclonal antibody directed against the CD20 antigen on B lymphocytes [23].

### 3.6. Predictive Factors for Relapse

For patients with relapsed AIP, maintenance therapy with steroids should be given with longer duration and higher dose than that of the initial maintenance therapy. Furthermore, pancreatic stones may form in some relapsing AIP patients, which might be induced by pancreatic juice stasis from incomplete obstruction of an irreversibly damaged pancreatic duct system [24]. Therefore, relapse of AIP should be avoided as much as possible. Identification of risk factors for relapse may help to identify high-risk patients who would benefit from maintenance therapy up front, and allow short-term therapy in lower-risk patients who may not need long-term therapy.

Hirano et al. [25] reported that obstructive jaundice is a predictive factor for unfavorable events. Kubota et al. [8] reported that diffuse pancreatic swelling independently predicted a relapse of AIP. Raina et al. described that relapse occurred in 7 of 9 patients (78%) with extrapancreatic biliary stenosis after withdrawal of immunosuppressive therapy [21]. Ghazale et al. reported that the relapse rate (64%) of patients with proximal biliary stenosis was significantly higher than that of patients with distal biliary stenosis alone (32%) [22]. In Korean data, relapse rate in patients with intrahepatic or proximal biliary stenosis was 65% compared with 25% in those without proximal biliary disease [26]. In a multicenter study [17], the relapse rate of AIP was significantly higher in patients with persistent elevation of serum IgG4 levels (30%, 34/115) than in those with normalized serum IgG4 levels (10%, 7/69). Although serum IgG4 levels fluctuated by more than 30 mg/dL in 94 of 172 patients (55%) during maintenance therapy, reelevation of serum IgG4 levels was detected in 37 of 54 patients (69%) who relapsed during maintenance therapy. The presence of proximal bile duct stenosis and elevated serum IgG4 levels may be predictive factors of relapse of AIP. Changes in serum IgG4 levels during serial checkups may provide clinically useful information to detect relapse of AIP earlier.

In the future, it will be necessary to verify the validity of the Japanese regimen of steroid therapy for AIP. The necessity, drugs, and duration of maintenance therapy need to be clarified by prospective studies.

As recent interesting reports have shown that pancreatic cancer was complicated with AIP [27, 28], we have to pay attention not only AIP but also pancreatic cancer even in a follow-up period.

## 4. Conclusion

It is important to differentiate AIP from pancreatic cancer before starting therapy. Steroids are the standard therapy for AIP, but this regimen should be evaluated in prospective studies.
